# Glomerular plasmalemma vesicle‐associated protein‐1 as an endothelial remodelling marker complementing C4d in chronic active antibody‐mediated rejection

**DOI:** 10.1111/his.70162

**Published:** 2026-04-21

**Authors:** Yuto Igarashi, Mayu Shimokawa, Kunio Kawanishi, Toshihito Hirai, Hiroshi Seino, Tomokazu Shimizu, Hideki Ishida, Toshio Takagi

**Affiliations:** ^1^ Department of Urology Tokyo Women's Medical University Tokyo Japan; ^2^ Department of Anatomy Showa Medical University School of Medicine Tokyo Japan; ^3^ Internal Medicine (Nephrology) Showa Medical University Fujigaoka Hospital Yokohama Japan; ^4^ Department of Experimental Pathology, Institute of Medicine University of Tsukuba Tsukuba Ibaraki Japan; ^5^ Division of Pathology, Kidney Center Tokyo Women's Medical University Tokyo Japan; ^6^ Department of Organ Transplant Medicine Tokyo Women's Medical University Tokyo Japan; ^7^ Present address: Department of Colorectal Surgery Kansai Medical University Hirakata Osaka Japan

**Keywords:** antibody‐mediated rejection, C4d, endothelial remodelling, kidney transplantation, PLVAP/PV‐1, transplant glomerulopathy

## Abstract

**Aims:**

Chronic active antibody‐mediated rejection (caABMR) is a major cause of late kidney allograft failure, yet reliable histological indicators of microvascular injury and therapeutic response remain insufficient. Complement C4d deposition reflects complement activation but does not fully capture endothelial remodelling, particularly in ABO‐incompatible transplantation. Plasmalemma vesicle‐associated protein‐1 (PV‐1), encoded by PLVAP and normally absent from glomerular endothelium, is upregulated during endothelial remodelling. We investigated whether glomerular PV‐1 complements C4d in identifying active endothelial injury in caABMR.

**Methods and results:**

A total of 429 allograft biopsies from 126 patients with caABMR and 345 surveillance biopsies from 94 stable recipients were analysed. Glomerular PV‐1 and C4d immunofluorescence intensities were quantified and correlated with histopathological lesions, renal function, and donor‐specific antibodies, with stratification by ABO compatibility. Both PV‐1 and C4d were associated with microvascular inflammation and transplant glomerulopathy. While C4d reflected cumulative complement activation and showed staining in ABO‐incompatible grafts, PV‐1 specifically identified endothelial remodelling, particularly in lesions with double‐contour formation, and demonstrated dynamic reduction following B‐cell‐directed therapy. Low‐vacuum scanning electron microscopy correlated with the presence of de novo PV‐1 expression in glomerular endothelial cells. In multivariable Cox models incorporating ΔPV‐1 and ΔC4d with clinical covariates, ΔPV‐1 remained independently associated with death‐censored graft survival, whereas ΔC4d did not.

**Conclusions:**

Glomerular PV‐1 functions as a dynamic marker of endothelial remodelling that complements the static C4d footprint of complement activation. Combined assessment of PV‐1 and C4d captures distinct dimensions of microvascular pathology and may refine histopathological evaluation of injury and treatment response in caABMR.

## Graphical abstract

Glomerular plasmalemma vesicle‐associated protein‐1 (PV‐1/PLVAP) identifies dynamic endothelial remodelling in chronic active antibody‐mediated rejection (caABMR), complementing glomerular C4d, which reflects a static footprint of complement activation. Combined assessment of PV‐1 and C4d captures complementary dimensions of microvascular pathology and refines histopathologic evaluation of treatment response in kidney allografts.

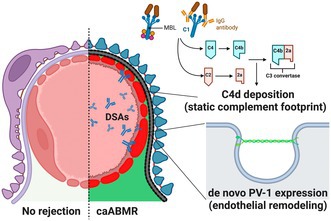

**Abbreviations**
C4d: complement 4d
caABMR: chronic active antibody‐mediated rejection
DFPP: double‐filtration plasmapheresis
DM: diabetes mellitus
DSA: donor‐specific antibody
DSG: deoxyspergualin
eGFR: estimated glomerular filtration rate
FSGS: focal segmental glomerulosclerosis
HLA: human leucocyte antigen
IF: immunofluorescence
IVIg: intravenous immunoglobulin
LM: light microscopy
LVSEM: low‐vacuum scanning electron microscopy
MPGN: membranoproliferative glomerulonephritis
mPSL: methylprednisolone
MVI: microvascular inflammation
PGNMID: proliferative glomerulonephritis with monoclonal immunoglobulin deposits
PIGN: post‐infectious glomerulonephritis
PTC: peritubular capillary
PV‐1: plasmalemma vesicle‐associated protein‐1
sCr: serum creatinine
TCMR: T cell‐mediated rejection
TG: transplant glomerulopathy
UP: urinary protein

## Introduction

Antibody‐mediated rejection (ABMR) remains one of the leading causes of late allograft failure after kidney transplantation. Increasing evidence indicates that both complement activation and chronic endothelial injury contribute to the pathogenesis and progression of ABMR.^1^ Donor‐specific antibodies (DSA), microvascular inflammation, and transplant glomerulopathy (TG) serve as key diagnostic and prognostic features in this setting.^2^, ^3^

The Banff classification has continually evolved in response to these insights. The introduction of C4d‐negative ABMR in Banff 2013 and further refinements in Banff 2017 clarified that alloantibody‐mediated injury can occur in the absence of classical complement pathway deposition.^4^, ^5^ Although peritubular capillary (PTC) C4d staining remains a hallmark of complement fixation, its diagnostic and prognostic performance is heterogeneous; the absence of PTC C4d does not exclude ABMR, and focal versus diffuse staining may carry distinct clinical implications.^6^, ^7^

Beyond PTC‐based assessment, attention has increasingly shifted towards glomerular C4d, which has been identified in chronic alloimmune injury and structural endothelial remodelling. Several investigations have demonstrated glomerular C4d positivity in chronic TG and thrombotic microangiopathy (TMA), where it reflects capillary wall remodelling rather than purely active complement fixation.^8^, ^9^, ^10^ These observations indicate that glomerular C4d may provide complementary information about chronic microvascular perturbation that is not captured by PTC C4d scoring alone.

In parallel, recent developments in clinical practice have broadened the immunologic landscape of kidney transplantation. ABO‐incompatible transplantation – historically considered prohibitive – has become an increasingly routine procedure across many transplant programmes worldwide with outcomes approaching those of ABO‐compatible transplantation, following the advancement of desensitization protocols and post‐transplant immunomodulation (e.g., accommodation).^11^ Such diverse alloimmune environments highlight the need for biomarkers that differentiate static complement footprints from dynamic endothelial remodelling.

Plasmalemmal vesicle‐associated protein‐1 (PV‐1), encoded by *PLVAP*, is a structural component of endothelial diaphragms and is normally absent in glomerular capillaries.^12^ De novo PV‐1 expression has been documented in TG,^13^ and in immune‐mediated proliferative glomerulopathies with monoclonal IgG deposits and/or lupus nephritis, supporting its role as a marker of glomerular endothelial remodelling.^14^ Taken together, these observations suggest that PV‐1 may serve as a sensitive readout of fenestral remodelling and endothelial barrier disruption under several conditions, including chronic alloimmune stress.

However, the clinical and prognostic significance of PV‐1 in ABMR has not been systematically evaluated, nor has its relationship to glomerular C4d been clarified in large‐scale biopsy cohorts encompassing heterogeneous immunologic backgrounds.

Therefore, this study comprehensively assessed glomerular C4d and PV‐1 expression in a well‐characterized cohort of chronic active ABMR, examining their histopathologic associations, clinical determinants, and prognostic impact. We aimed to determine whether PV‐1 functions as a dynamic endothelial remodelling marker that complements the static information provided by C4d, thereby refining morphologic evaluation and risk stratification in chronic active ABMR.

## Methods

### Patients and Study Approval

This single‐centre retrospective cohort study was approved by the Ethics Committee of Tokyo Women's Medical University (Approval No. 2024‐0128) and conducted in accordance with the Declaration of Helsinki. Consecutive kidney allograft biopsies performed between January 2012 and December 2022 were screened. Because of the retrospective nature of the study, informed consent was obtained using an opt‐out approach, as approved by the institutional review board. Biopsies with recurrent or de novo primary glomerular disease, BK virus nephropathy, or other non‐alloimmune causes of glomerular endothelial injury were excluded as predefined criteria (details in Data S1).

### Study Design and Patient Selection

This study was designed as a single‐centre retrospective cohort analysis. Kidney allograft biopsies fulfilling Banff‐defined criteria for antibody‐mediated rejection (ABMR) were included. Chronic active ABMR was defined according to the 2022 Banff classification, requiring evidence of chronic tissue injury together with ongoing antibody‐mediated activity. Surveillance biopsies from recipients with stable graft function and no histological evidence of rejection or BK virus nephropathy served as controls. Detailed inclusion and exclusion criteria are described in the Data S1.

### Clinical Data Collection

Clinical data were extracted from electronic medical records and included recipient and donor demographics, transplant characteristics, ABO compatibility, HLA mismatch, donor‐specific antibody (DSA) status, renal function parameters, and anti‐rejection therapies. Graft failure was defined as return to dialysis. Details of immunosuppressive regimens and treatment protocols are provided in Data S1.

### Pathologic Evaluation

All biopsy specimens were evaluated using standard light microscopy and immunofluorescence according to the 2022 Banff classification.^15^ Routine histologic stains included haematoxylin–eosin, periodic acid–Schiff, Masson's trichrome, and periodic acid–methenamine silver.

### Immunofluorescence Staining and Quantitative Analysis of Transplant Kidney Biopsies

Frozen sections were stained for C4d and PV‐1 using monoclonal primary antibodies and Alexa Fluor–conjugated secondary antibodies. Images were acquired under standardized conditions and quantified in ImageJ. Glomerular intensity was measured in predefined whole‐tuft ROIs and expressed as background‐subtracted integrated density per area (IntDen/Area). Antibodies, imaging settings, and reproducibility are detailed in Data S1.

### Multicolor Immunofluorescence and Ultrastructural Analysis

Selected formalin‐fixed paraffin‐embedded sections were subjected to multicolor immunofluorescence for endothelial and glycocalyx‐associated markers. Ultrastructural features of glomerular basement membranes (GBMs) were assessed using modified periodic acid–methenamine silver staining and low‐vacuum scanning electron microscopy to corroborate endothelial remodelling.^16^ Technical details are described in Data S1.

### Statistical Analysis

Continuous variables were analysed using parametric or non‐parametric tests as appropriate. Associations between clinical variables and glomerular staining intensity were evaluated using logistic regression models. Graft survival was analysed by Kaplan–Meier methods with log‐rank testing. Death‐censored graft survival was additionally evaluated using multivariable Cox proportional hazards models incorporating ΔPV‐1 and ΔC4d together with prespecified clinical covariates. Details of covariates, handling of repeated biopsies, proportional hazards diagnostics, and sensitivity analyses are provided in the Data S1.

## Results

### Patient Characteristics and Biopsy Cohort

Among 3826 transplant biopsies performed during the study period, 429 biopsies from 126 patients met criteria for chronic active antibody‐mediated rejection (caABMR) and were included for analysis (Figure S1). A control cohort comprised 345 surveillance biopsies from 94 stable recipients with long‐term follow‐up. Baseline clinical characteristics, including recipient age, post‐transplant duration, renal function, and the proportion of ABO‐incompatible transplantation are summarized in Table 1.

**Table 1:** Clinical characteristics of patients with antibody‐mediated rejection versus control groups

Characteristics	ABMR (*n* = 126)	Control (*n* = 94)	*P*‐value
Recipient age (years)	53.5 (44.7–64.0)	46.0 (38.0–61.0)	0.0032
Donor age (years)	60.0 (53.0–65.0)	61.0 (52.8–66.0)	0.608
Male recipient (%)	62.0	68.1	0.3935
Months post‐transplantation	30.5 (12.0–63.8)	50.0 (34.8–69.8)	0.0011
ABO incompatibility (%)	34.1	24.5	0.1383
Total number of HLA mismatches	3.0 (3.0–5.0)	—	—
De novo DSA (%)	34.4	—	—
Diabetes (%)	25.0	22.3	0.7487
MP (%)	62.7	—	—
IVIg (%)	57.1	—	—
Rituximab (%)	56.3	—	—
DFPP (%)	23.8	—	—
DSG (%)	7.9	—	—
Last eGFR (mL/min/1.73 m^2^)	33.3 (16.9–43.9)	40.8 (33.3–50.4)	<0.0001

Values are presented as medians (interquartile ranges). — indicates not applicable.

Abbreviations: DFPP, double‐filtration plasmapheresis; DSA, donor‐specific antibody; DSG, deoxyspergualin; eGFR, estimated glomerular filtration rate; HLA, human leucocyte antigen; IVIg, intravenous immunoglobulin; MP, methylprednisolone.

Control recipients were stringently defined as long‐term stable cases with ≥3 biopsies over >8 years and no histological evidence of rejection or BK virus nephropathy (Figure S1).

### Histopathological Features of caABMR and Transplant Glomerulopathy

**Figure 1:**
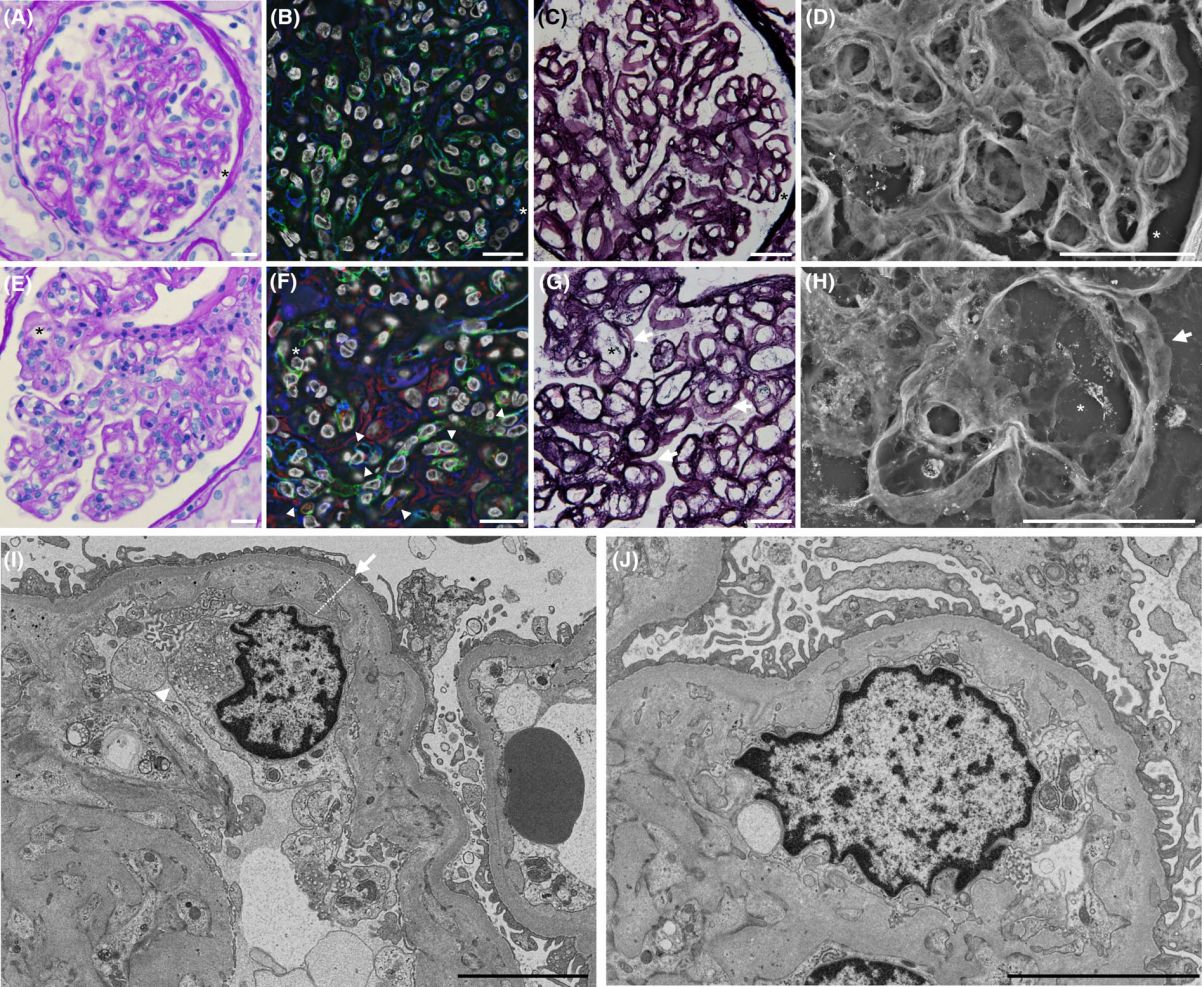
De novo PV‐1 expression and ultrastructural features of transplant glomerulopathy. (**A–D**) Protocol biopsy obtained 814 days post‐transplantation showing preserved glomerular architecture without evidence of glomerular basement membrane (GBM) duplication. (**E–H**) Index biopsy obtained 2,110 days post‐transplantation (ABO‐compatible) showing transplant glomerulopathy (TG) with GBM double contours and subendothelial widening. (**A, E**) Periodic acid–Schiff (PAS) staining. (**B, F**) Multicolor immunofluorescence for CD31 (green), PV‐1 (red), and Sambucus nigra lectin (SNA; blue). SNA was used as an endothelial/glycocalyx‐associated reference marker to support anatomic localization of PV‐1 along glomerular capillary walls. Arrowheads indicate de novo PV‐1 expression in glomerular capillary endothelial cells, and arrows indicate double‐contour formation. (**C, G**) Modified periodic acid–methenamine silver (PAM) staining showing intact GBM in the protocol biopsy and segmental‐to‐diffuse double contours in TG. (**D, H**) Corresponding low‐vacuum scanning electron microscopy images demonstrating capillary wall remodelling with subendothelial expansion. (**I, J**) Representative transmission electron microscopy (TEM) images confirming ultrastructural features of TG, including GBM duplication (arrows and dotted lines) and endothelial injury with increased vesicular/caveola‐like profiles (arrowheads). TEM images are shown for qualitative confirmation and were not included in quantitative analyses.

Protocol biopsies from control recipients showed preserved glomerular architecture without endothelial remodelling. In contrast, caABMR biopsies demonstrated mesangial expansion, GBM duplication, and ultrastructural features of TG. Multicolor immunofluorescence and ultrastructural analyses demonstrated findings consistent with de novo glomerular PV‐1 expression localized to fenestrated endothelial cells in TG lesions (Figure 1).

**Figure 2:**
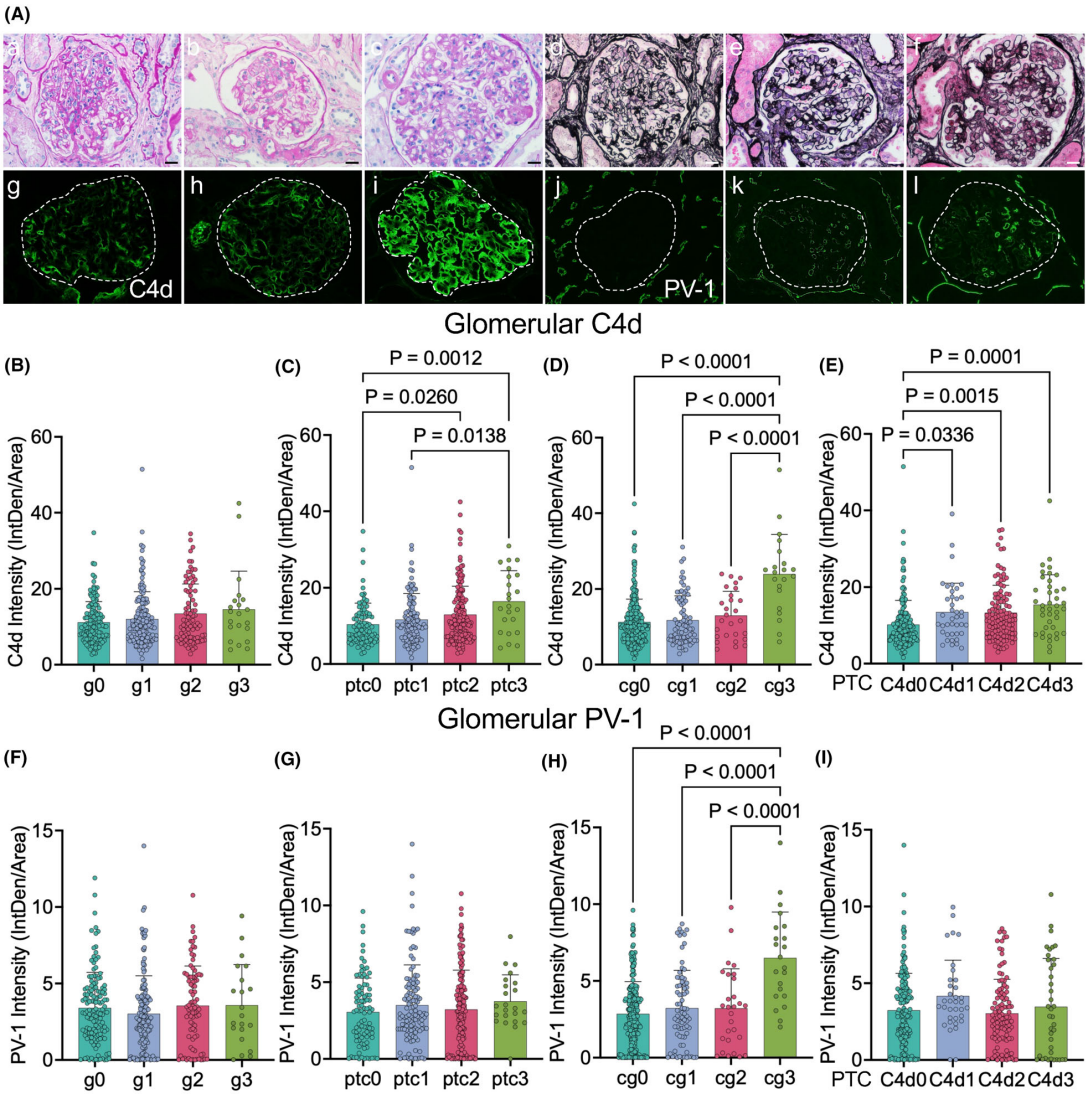
Glomerular C4d deposition and PV‐1 expression in chronic active ABMR. (**A**) Representative images of allograft biopsies from a patient with chronic active antibody‐mediated rejection (caABMR). Panels (a–c) show PAS staining; (d–f) PAM staining; (g–i) immunofluorescence for glomerular C4d; and (j–l) immunofluorescence for glomerular PV‐1. At baseline (a, d, g, j), PV‐1 is absent from glomerular endothelium despite the presence of chronic glomerulopathy (cg) lesions. At 4 years post‐transplant (b, e, h, k), segmental cg lesions are visible with PAM staining and PV‐1 becomes focally detectable. At 5 years 9 months (c, f, i, l), focal‐to‐diffuse cg lesions are present with corresponding diffuse PV‐1 induction in glomerular capillary loops. Scale bars: 20 μm. (**B–D**) Quantitative glomerular C4d intensity (IF‐based) stratified by Banff glomerulitis (g), peritubular capillaritis (ptc), and transplant glomerulopathy (cg). (**E**) Relationship between glomerular C4d intensity and the Banff peritubular capillary (PTC) C4d score (C4d0–C4d3). (**F–H**) Quantitative glomerular PV‐1 intensity stratified by Banff g, ptc, and cg categories. (**I**) Glomerular PV‐1 intensity across Banff PTC‐C4d scores.

Quantitative immunofluorescence demonstrated significantly increased glomerular C4d and PV‐1 intensities in caABMR compared with controls (Figure 2A). Glomerular C4d intensity correlated with peritubular capillaritis, TG, and Banff PTC C4d scores, whereas PV‐1 expression correlated selectively with cg score, consistent with chronic endothelial remodelling (Figure 2B–H). Representative longitudinal control biopsies further illustrated that PV‐1 is consistently absent in stable glomeruli, whereas low‐level glomerular C4d can be detectable even in controls depending on immunological context (Figures S2 and S3). Representative serial biopsies demonstrating the spectrum of glomerular PV‐1 intensity and its longitudinal change following anti‐rejection therapy are provided in Figure S4.

### Stratified Analyses by Immunological and Clinical Context

Both glomerular C4d and PV‐1 were higher in late‐phase (≥1 year) caABMR biopsies with microvascular inflammation (MVI; Banff g + ptc ≥2) than in controls (Figure S5A,B). In early‐phase biopsies (<1 year), only C4d showed a significant increase (Figure S5A). In ABO‐incompatible grafts, stable controls showed low‐level glomerular C4d detectable under standardized acquisition settings, whereas PV‐1 remained absent in controls and preferentially elevated in caABMR (Figure S5C,D). PV‐1 intensity did not show a consistent association with contemporaneous DSA status (Figure S5F). Stratification by diabetes mellitus (DM) status showed that C4d, but not PV‐1, differed significantly according to DM, supporting distinct clinical determinants for these markers (Figure S5G,H).

### Association of C4d and PV‐1 with Renal Function and Prognostic Performance

**Figure 3:**
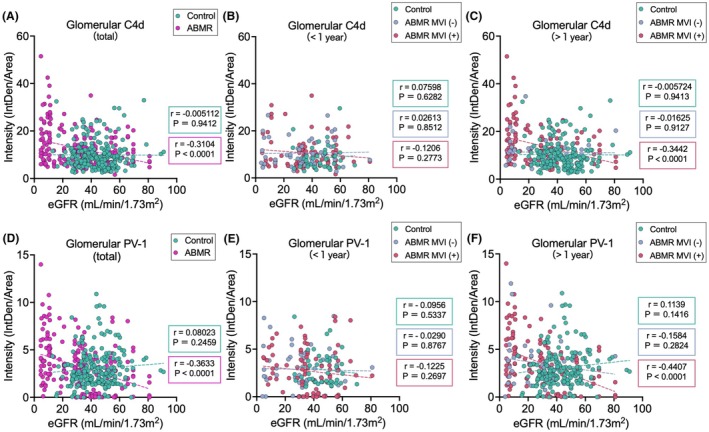
Correlation between glomerular C4d and PV‐1 intensity and renal function in chronic active antibody‐mediated rejection (ABMR). Scatter plots show correlations between renal function, assessed by estimated glomerular filtration rate (eGFR), and the intensity of glomerular C4d and plasmalemma vesicle‐associated protein‐1 (PV‐1) staining in chronic active ABMR and control biopsies, with additional stratification of ABMR cases by the presence or absence of microvascular inflammation (MVI). Panels (**A–C**) depict glomerular C4d, while panels (**D–F**) depict glomerular PV‐1. Panels (**B, E**) represent biopsies obtained within 1 year after transplantation, whereas panels (**C, F**) represent biopsies obtained more than 1 year post‐transplant. Pearson's correlation coefficients (r) and *P* values are indicated in each panel. [Colour figure can be viewed at wileyonlinelibrary.com]

Based on these histopathological observations, the next step was to assess the clinical relevance of glomerular C4d and PV‐1 by analysing their associations with renal function and prognostic performance (Figure 3A–F). In the overall cohort, both markers showed inverse correlations with eGFR (Figure 3A,D). Stratification by post‐transplant phase revealed that this relationship was confined to late‐phase biopsies obtained ≥1 year post‐transplant (Figure 3C,F), whereas no significant associations were observed in early‐phase biopsies (Figure 3B,E). The inverse correlations were most pronounced in MVI‐positive cases, underscoring the impact of microvascular injury on progressive functional decline.

### Dynamic Changes in Glomerular Markers and Graft Survival

**Figure 4:**
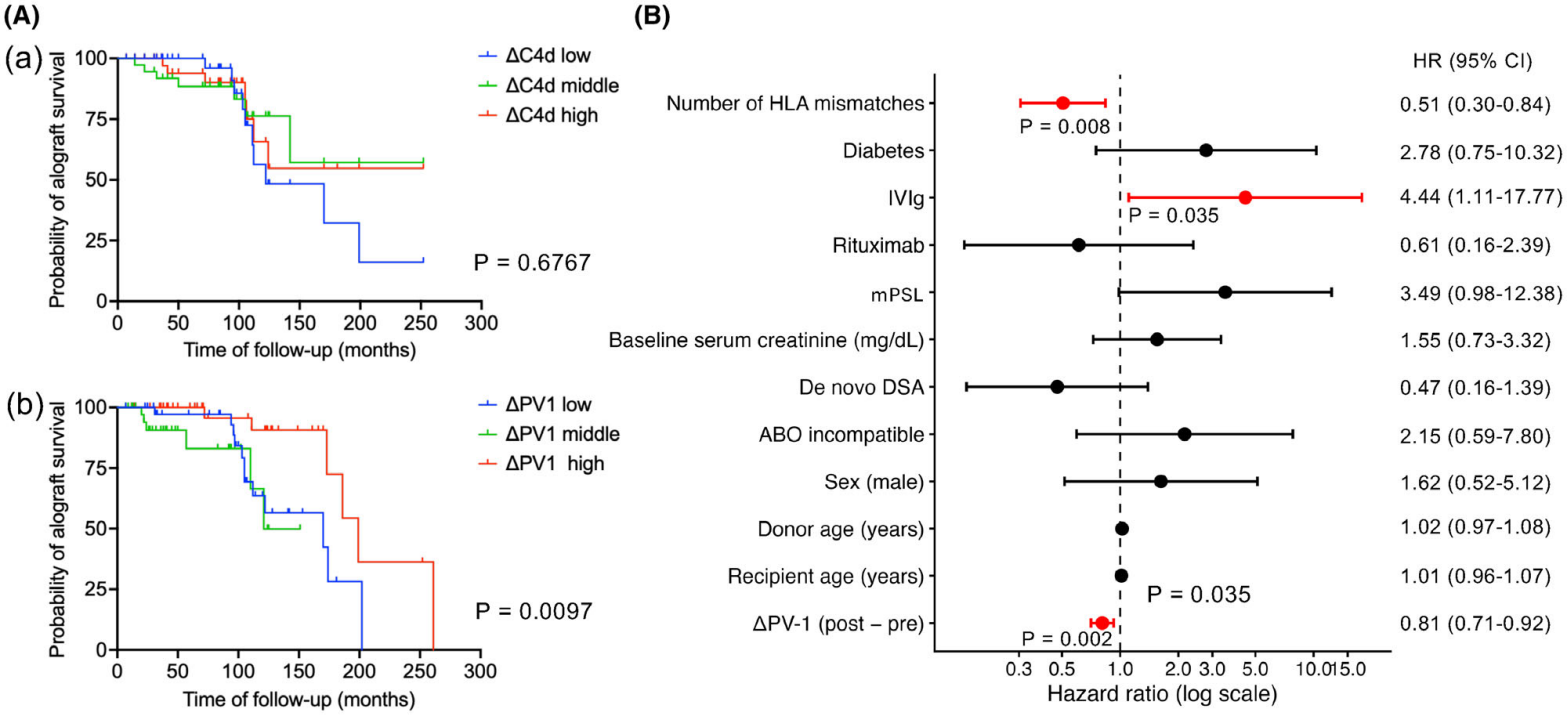
Changes in PV‐1, but not C4d, are associated with death‐censored graft failure. (**A**) (a) Kaplan–Meier analysis of death‐censored graft survival stratified by tertiles of ΔC4d (post‐treatment – pretreatment). No significant difference in graft survival was observed among the three groups (log‐rank test). (b) Kaplan–Meier analysis stratified by tertiles of ΔPV‐1 (post–pre) demonstrated significant differences in death‐censored graft survival (log‐rank test). (**B**) Multivariable Cox proportional hazards model including ΔPV‐1 and ΔC4d (both defined as post‐treatment – pretreatment) together with clinical covariates (age, sex, ABO incompatibility, de novo DSA, steroid pulse therapy, rituximab, and IVIg). Hazard ratios (HRs) and 95% confidence intervals (CIs) are shown. Variables with *P* < 0.05 are highlighted in red. [Colour figure can be viewed at wileyonlinelibrary.com]

To evaluate treatment responsiveness, within‐patient changes in glomerular C4d and PV‐1 intensities were analysed as Δ values (Δ = post‐treatment − pretreatment; Figure 4). In Kaplan–Meier analyses using tertiles of ΔC4d, no significant difference in death‐censored graft survival was observed (Figure 4A). In contrast, tertiles of ΔPV‐1 showed significant separation of graft survival curves (Figure 4A), indicating that larger decreases in PV‐1 intensity (more negative ΔPV‐1) were associated with improved outcomes; the clinical characteristics of each tertile are summarized in Table 2.

**Table 2:** Clinical characteristics among ABMR patients stratified by ΔPV‐1 tertiles

Characteristics	ΔPV‐1 low (*n* = 40)	ΔPV‐1 middle (*n* = 40)	ΔPV‐1 high (*n* = 41)	*P*‐value
ΔPV‐1	−2.77 (−3.39 to −2.14)	0.23 (−0.36 to 0.11)	2.97 (2.48 to 3.45)	<0.0001
Recipient age (years)	52.3 (48.6–55.9)	56.9 (53.5–60.3)	52.7 (48.9–56.4)	0.1775
Donor age (years)	58.3 (55.0–61.7)	59.8 (56.8–62.7)	58.7 (55.5–61.7)	0.9341
Male recipient (%)	57.5	62.5	65.8	0.7388
Months post‐transplantation	51.5 (32.9–69.3)	22.8 (15.6–30.2)	67.1 (48.1–86.1)	<0.0001
ABO incompatibility (%)	43.9	37.5	43.9	0.0561
Total number of HLA mismatches	3.6 (3.0–4.1)	3.9 (3.4–4.3)	3.5 (3.1–3.9)	0.3022
De novo DSA (%)	40.0	25.0	37.5	0.3073
Diabetes (%)	22.5	17.5	32.5	0.2839
MP (%)	67.5	65.0	60.9	0.8257
IVIg (%)	55.0	65.0	56.1	0.6051
Rituximab (%)	55.0	50.0	68.3	0.2207
DFPP (%)	25.0	20.0	29.3	0.6244
DSG (%)	7.5	10.0	7.3	0.8906
Last eGFR (mL/min/1.73 m^2^)	27.5 (21.5–33.5)	33.1 (28.0–38.2)	34.6 (28.8–40.4)	0.1503

Abbreviations: DFPP, double‐filtration plasmapheresis; DSA, donor‐specific antibody; DSG, deoxyspergualin; eGFR, estimated glomerular filtration rate; HLA, human leucocyte antigen; IVIg, intravenous immunoglobulin; MP, methylprednisolone.

We next performed a multivariable Cox proportional hazards model incorporating ΔPV‐1 and ΔC4d simultaneously together with clinical covariates (age, sex, ABO incompatibility, de novo DSA, steroid pulse therapy, rituximab, and IVIg; Figure 4B). In this model, ΔPV‐1 remained independently associated with death‐censored graft survival, whereas ΔC4d did not demonstrate an independent association. Variables with *P* < 0.05 are highlighted in the forest plot (Figure 4B).

Exploratory analyses of clinical determinants of ΔPV‐1 and subgroup survival analyses stratified by ABO compatibility and rituximab exposure are provided in Figures S6–S9. Kaplan–Meier analyses stratified by individual treatment modalities (mPSL, DFPP, DSG, IVIg, and rituximab) are shown in Figure S6. Multivariable logistic regression analyses identifying clinical covariates associated with ΔC4d and ΔPV‐1 are provided in Figure S7. Subgroup Kaplan–Meier analyses stratified by ABO compatibility and rituximab exposure are shown in Figures S8 and S9. A sensitivity Cox model excluding ΔC4d (Model 1) yielded consistent results for ΔPV‐1 (Figure S10 and Table S1).

## Discussion

This study demonstrates that glomerular C4d and plasmalemma vesicle‐associated protein‐1 (PV‐1) capture biologically distinct yet complementary dimensions of endothelial injury in chronic active antibody‐mediated rejection (caABMR). While glomerular C4d reflects cumulative alloantibody‐associated complement activation within capillary walls, PV‐1 highlights ongoing endothelial stress and remodelling, particularly in the setting of established TG. Although the Banff PTC C4d score remains central to ABMR diagnosis, its prognostic reliability is limited because its sensitivity is variable despite high specificity for ABMR.^6^ Previous reports have described conflicting associations between PTC C4d positivity and clinical outcomes: diffuse C4d staining has been linked to worse short‐term prognosis,^17^ whereas other studies observed modest or no long‐term prognostic impact.^6^, ^7^ The present findings refine this framework by demonstrating that maximum glomerular C4d intensity, assessed as a continuous variable and anatomically distinct from PTC scoring, provides prognostic value independent of categorical PTC scoring. In contrast, dynamic changes in glomerular C4d (ΔC4d) did not correlate with treatment response or graft survival, underscoring the limited utility of glomerular C4d as a dynamic biomarker.

Importantly, our study was intentionally restricted to caABMR and long‐term stable controls, and did not evaluate PV‐1 or glomerular C4d across other allograft injury categories such as T‐cell‐mediated rejection, acute thrombotic microangiopathy, acute ABMR, or TG without ABMR.

The association between rituximab therapy and declines in PV‐1 expression aligns with existing literature demonstrating that CD20‐directed therapy is more effective at reducing DSAs than IVIg‐based regimens alone. Rituximab has been shown to reduce HLA class I and II DSAs within several months and to stabilize graft function in chronic AMR.^18^

Importantly, this observation should not be interpreted as reversal of established structural lesions, but rather as attenuation of active endothelial stress superimposed on fixed chronic injury. However, the molecular mechanisms governing PV‐1 induction, maintenance, and resolution – particularly within the context of TG – require further mechanistic investigation.

Glomerular C4d deposition is a recognized marker of lectin‐pathway complement activation and is encountered across a broad range of primary glomerular diseases. Glomerular C4d deposition has been reported across multiple immune‐mediated and complement‐associated glomerulopathies,^19^, ^20^ reinforcing its diagnostic utility while underscoring the need for contextual interpretation in transplant biopsies. By contrast, PV‐1 shows far more restricted expression across native kidney diseases. Recent studies—including findings from proliferative GN with monoclonal IgG deposits and lupus nephritis – have demonstrated de novo PV‐1 expression in endothelial injury–associated glomerulopathies,^14^ supporting its greater specificity as a marker of active endothelial remodelling. In the transplant setting, this restricted expression pattern positions PV‐1 as a useful adjunct for differentiating alloimmune‐mediated injury from recurrent or de novo primary disease.

Several limitations warrant consideration. This single‐centre study limited the power of subgroup analyses, particularly for longitudinal (Δ‐based) metrics. Because treatment allocation was not randomized, confounding by indication – especially regarding corticosteroid pulse therapy – cannot be completely excluded. Δ‐based analyses may also be affected by baseline lesion severity and biopsy interval variability. The control cohort, deliberately restricted to long‐term stable recipients with multiple protocol biopsies, improves internal validity but may limit generalizability. Finally, neither C4d nor PV‐1 is entirely disease‐specific; C4d is detected across multiple primary glomerulopathies, whereas PV‐1 may be upregulated in select proliferative diseases. Thus, both markers must be interpreted within the broader clinical and histopathologic context.

Despite these limitations, the present findings establish glomerular C4d and PV‐1 as complementary biomarkers of chronic alloimmune injury. While C4d reflects cumulative complement activation, PV‐1 captures dynamic endothelial remodelling with prognostic and therapeutic relevance. PV‐1 may therefore serve not only as a prognostic indicator but also as a treatment‐responsive marker suitable for risk stratification and therapeutic monitoring in caABMR. Larger multicentre cohorts and mechanistic studies will be essential to validate these findings and to refine biomarker‐guided approaches for the diagnosis and management of ABMR.

## Author contributions

K.K. conceptualized the study. Y.I. and M.S. contributed to the methodology, formal analysis, and original draft preparation. H.S. (medical laboratory technologist) performed the transmission electron microscopy analyses and contributed to ultrastructural interpretation during the revision. K.K., T.H., T.S., H.I., and T.T. provided resources. T.H., H.S., T.S., and H.I. conducted the review and editing. Supervision was performed by K.K. and T.T. Funding acquisition: K.K. All authors reviewed and approved the final version of the manuscript.

## Funding information

This study was supported by a Japan Science and Technology Agency (JST) START University Promotion Type Grant (JPMJST2052), awarded to KK.

## Conflict of interest statement

The authors declare no conflict of interest.

## Supporting information


**Figure S1.** Flowchart of patient selection for the study.
**Figure S2.** Glomerular C4d deposition and PV‐1 expression in a control case with ABO compatibility.
**Figure S3.** Glomerular C4d deposition and PV‐1 expression in a control case with ABO incompatibility.
**Figure S4.** Representative longitudinal glomerular C4d and PV‐1 immunofluorescence images in caABMR cases spanning low, intermediate, and high PV‐1 intensity.
**Figure S5.** Glomerular C4d deposition and PV‐1 expression in relation to clinical and histological factors in chronic active antibody‐mediated rejection.
**Figure S6.** Survival curves stratified by treatment modalities in chronic active antibody‐mediated rejection.
**Figure S7.** Multivariable logistic regression analyses of clinical factors associated with within‐patient changes (Δ) in glomerular C4d and PV‐1 intensity.
**Figure S8.** Allograft survival according to dynamic changes in glomerular C4d and PV‐1 intensity stratified by ABO compatibility.
**Figure S9.** Death‐censored graft survival according to dynamic changes in glomerular C4d and PV‐1 intensity stratified by rituximab exposure.
**Figure S10.** Sensitivity multivariable Cox proportional hazards model (Model 2) incorporating ΔPV‐1 and ΔC4d for death‐censored graft failure.

**Table S1.** Multivariable Cox proportional hazards models and proportional hazards diagnostics (Schoenfeld residual tests).

## Data Availability

The data supporting the findings of this study are available within the article and its Supporting Information—S1.
