# Supplementary material for: Glomerular plasmalemma vesicle‐associated protein‐1 as an endothelial remodelling marker complementing C4d in chronic active antibody‐mediated rejection
Source: Histopathology. 2026 Apr 21;89(2):372–82. doi: 10.1111/his.70162 (PMC13341019; doi:10.1111/his.70162)
Supplement: Supplementary file 1 — Figure S1. Flowchart of patient selection for the study. Figure S2. Glomerular C4d deposition and PV‐1 expression in a control case with ABO compatibility. Figure S3. Glomerular C4d deposition and PV‐1 expression in a control case with ABO incompatibility. Figure S4. Representative longitudinal glomerular C4d and PV‐1 immunofluorescence images in caABMR cases spanning low, intermediate, and high PV‐1 intensity. Figure S5. Glomerular C4d deposition and PV‐1 expression in relation to clinical and histological factors in chronic active antibody‐mediated rejection. Figure S6. Survival curves stratified by treatment modalities in chronic active antibody‐mediated rejection. Figure S7. Multivariable logistic regression analyses of clinical factors associated with within‐patient changes (Δ) in glomerular C4d and PV‐1 intensity. Figure S8. Allograft survival according to dynamic changes in glomerular C4d and PV‐1 intensity stratified by ABO compatibility. Figure S9. Death‐censored graft survival according to dynamic changes in glomerular C4d and PV‐1 intensity stratified by rituximab exposure. Figure S10. Sensitivity multivariable Cox proportional hazards model (Model 2) incorporating ΔPV‐1 and ΔC4d for death‐censored graft failure. [file HIS-89-372-s002.docx]

**Supplementary Material for**

**Glomerular plasmalemma vesicle-associated protein-1 as an endothelial remodeling marker complementing C4d in chronic active antibody-mediated rejection**

Yuto Igarashi^1#^＾, Mayu Shimokawa^2,3#^, Kunio Kawanishi^2,4^*, Toshihito Hirai^1^, Hiroshi Seino^5^, Tomokazu Shimizu^1^, Hideki Ishida^1,6^, Toshio Takagi^1^

^1^Department of Urology, Tokyo Women’s Medical University Hospital, Tokyo, Japan

^2^Department of Anatomy, Showa Medical University School of Medicine, Hatanodai, Shinagawa-ku, Tokyo, Japan

^3^Internal Medicine (Nephrology), Showa Medical University Fujigaoka Hospital, Yokohama, Japan

^4^Department of Experimental Pathology, Institute of Medicine, University of Tsukuba, Tsukuba, Ibaraki, Japan

^5^Division of Pathology, Kidney Center, Tokyo Women’s Medical University, Tokyo, Japan

^6^Department of Organ Transplant Medicine, Tokyo Women’s Medical University, Tokyo, Japan.

*Correspondence: Kunio Kawanishi ([kukawanishi@med.showa-u.ac.jp](mailto:kukawanishi@med.showa-u.ac.jp)), Department of Anatomy, Showa Medical University School of Medicine, 1-5-8 Hatanodai, Shinagawa-ku, Tokyo, Japan, 142-8555

^#^YI and MS contributed equally to this work.

＾Current address: Department of Colorectal Surgery, Kansai Medical University, Shinmachi, Hirakata, Osaka, Japan

**This PDF file contains the Supplementary Methods, Figures S1–S10, and Table S1.**

**Supplementary Methods**

**Clinical data collection**

Clinical data were extracted from electronic medical records and included recipient age, sex, cause of end-stage kidney disease, donor age and type, ABO compatibility, number of HLA mismatches, presence of preformed or de novo donor-specific antibodies (DSA), urinary protein excretion, serum creatinine, and estimated glomerular filtration rate (eGFR). eGFR was calculated using the Japanese coefficient–adjusted equation.

Details of anti-rejection therapy were recorded for each biopsy episode, including methylprednisolone pulse therapy, intravenous immunoglobulin (IVIg), rituximab, double-filtration plasmapheresis (DFPP), and deoxyspergualin. Treatment selection and timing were determined by attending clinicians according to institutional practice. Death-censored graft failure was defined as return to maintenance dialysis, with death with a functioning graft treated as censoring. For Δ-based survival analyses restricted to patients with paired biopsies, follow-up time was measured from the index biopsy (pre-treatment biopsy) to graft failure or censoring. Δ values were calculated in cases with paired pre- and post-treatment biopsies, and were used as longitudinal response variables in downstream models.

**Histopathologic evaluation**

All biopsy specimens were evaluated using standard light microscopy and immunofluorescence. Routine histologic stains included hematoxylin–eosin, periodic acid–Schiff, Masson’s trichrome, and periodic acid–methenamine silver. Banff lesion scores were assigned according to the 2022 Banff classification by experienced renal pathologists blinded to clinical data. Discrepancies were resolved by consensus review. Biopsies were excluded if recurrent or de novo primary glomerular disease, BK virus nephropathy, or other non-alloimmune causes of glomerular endothelial injury were diagnosed on clinicopathologic correlation. Exclusion diagnoses included immune complex–mediated glomerulonephritis (e.g., IgA nephropathy, membranous nephropathy, lupus nephritis, membranoproliferative glomerulonephritis), infection-related glomerulonephritis, and monoclonal immunoglobulin–associated glomerulopathies.

**Immunofluorescence staining for C4d and PV-1**

C4d and PV-1 immunofluorescence were performed on separate 3-μm serial frozen sections (single-plex staining) using standardized protocols to avoid signal overlap and to maintain identical acquisition settings for each target. When both markers were analyzed for the same biopsy, ROIs were drawn on corresponding glomeruli whenever serial sections allowed clear anatomic matching; otherwise, the first 1–5 nonsclerotic glomeruli were quantified independently for each marker following the same serpentine rule. Sections were air-dried and incubated for 1 hour at room temperature with mouse monoclonal anti-human C4d antibody (Quidel, A213; 1:200 dilution) or mouse monoclonal anti–plasmalemma vesicle-associated protein-1 (PV-1) antibody (Abcam, ab8086; 1:20 dilution). After washing with phosphate-buffered saline (PBS), sections were incubated for 1 hour with Alexa Fluor 488–conjugated donkey anti-mouse IgG secondary antibody (Invitrogen, A21202; 1:500 dilution). Slides were mounted using anti-fade medium. Negative controls (secondary antibody only) were included in each staining run to confirm the absence of nonspecific fluorescence.

Fluorescence images were acquired using an Olympus AX80 immunofluorescence microscope equipped with a DP73 digital camera. Exposure time, gain, and illumination settings were fixed for all samples within each staining batch to ensure comparability. To minimize batch-to-batch variability, all images were acquired using identical microscope settings within each staining run, and background subtraction was applied uniformly as described below.

**Region of interest definition and quantitative analysis**

Quantitative analysis of glomerular staining was performed using ImageJ software (NIH, Bethesda, MD, USA). Regions of interest (ROIs) were defined a priori to encompass the entire glomerular tuft aligned to the Bowman’s space boundary. This approach was adopted to minimize bias associated with selective sampling of capillary loops or mesangial areas and to maintain consistency between C4d and PV-1 measurements.

Baseline mesangial C4d staining can be observed in both native and transplanted kidneys; therefore, no attempt was made to exclude mesangial regions within the glomerular ROI. For each biopsy, the first one to five nonsclerotic glomeruli encountered along a serpentine scanning path (systematically traversing the section in a fixed pattern) were analyzed to reduce field-selection bias. Background fluorescence was measured in adjacent non-glomerular areas and subtracted from raw intensity values. Intensity was quantified as background-subtracted integrated density per ROI area (IntDen/Area), an area-normalized metric equivalent to mean fluorescence intensity, and was used as the continuous intensity measure for downstream analyses. For each biopsy, intensity values were summarized as the mean IntDen/Area across analyzed glomeruli, and this mean value was used as the representative biopsy-level measurement.

**Definition of longitudinal change (ΔC4d and ΔPV-1)**

For patients with paired biopsies, ΔC4d and ΔPV-1 were defined as post-treatment minus pre-treatment intensity (Δ = post − pre), where the pre-treatment biopsy corresponded to the index biopsy at caABMR diagnosis and the post-treatment biopsy corresponded to the first follow-up biopsy performed after completion of anti-rejection therapy. Anti-rejection therapy was considered completed at the end of the planned institutional regimen for each episode (e.g., completion of steroid pulse, IVIg course, DFPP sessions, and/or rituximab administration), and the subsequent biopsy was treated as the post-treatment biopsy. For visualization, patients were categorized into tertiles (low/middle/high) based on the magnitude of Δ values.

The interval between paired biopsies was recorded for all cases, and sensitivity analyses incorporating biopsy interval as an additional covariate were performed (see Statistical analysis). If multiple follow-up biopsies were available, the earliest post-treatment biopsy with adequate tissue for quantitative analysis was used for Δ calculations to minimize time-dependent confounding.

**Reproducibility assessment**

Quantitative scoring was performed independently by two operators blinded to clinical information. Inter-observer reproducibility was assessed using intraclass correlation coefficients, which demonstrated high agreement for both C4d and PV-1 intensity measurements. Discrepant cases were reviewed jointly to confirm ROI placement and image quality. Intraclass correlation coefficients (ICCs) were calculated using a two-way random-effects model for absolute agreement, and 95% confidence intervals were reported.　ICCs were calculated on biopsy-level mean IntDen/Area values (averaged across 1–5 glomeruli per biopsy).

**Multicolor immunofluorescence**

Selected formalin-fixed paraffin-embedded (FFPE) sections were subjected to multicolor immunofluorescence to localize PV-1 expression within glomerular endothelial structures. Primary antibodies included mouse monoclonal anti-CD31 (DAKO, M082301-2), rabbit anti- PV-1 (PLVAP) (Novus Biologicals, NBP1-83911), and Sambucus nigra lectin (SNA) conjugated with Cy5. Secondary antibodies included Alexa Fluor 488–conjugated anti-mouse IgG and Alexa Fluor 568–conjugated anti-rabbit IgG (Invitrogen). Nuclei were counterstained with DAPI. Images were acquired using a Leica Thunder Imager.　FFPE sections were selected based on availability of paired biopsies and adequate tissue for parallel staining, prioritizing representative cases across the PV-1 intensity spectrum (negative/low/high) to illustrate staining patterns and potential interpretative pitfalls. SNA was included as an endothelial/glycocalyx-associated reference marker to support anatomic localization of PV-1 along glomerular capillary walls.

**Low-vacuum scanning electron microscopy**

Adjacent serial sections were stained using a modified periodic acid–methenamine silver method to visualize glomerular basement membrane architecture. Ultrastructural imaging was performed using a FlexSEM 1000 VP scanning electron microscope (Hitachi High-Tech Corporation, Japan) under variable-pressure (low-vacuum) conditions. Secondary electron, backscattered electron, and ultra-variable detectors were used to assess endothelial fenestration and basement membrane remodeling under low-vacuum conditions. Images were acquired at an accelerating voltage of 15 kV.

**Transmission electron microscopy**

Transmission electron microscopy (TEM) was performed in a representative caABMR case with established transplant glomerulopathy (Banff cg lesion) to confirm ultrastructural features of transplant glomerulopathy, including glomerular basement membrane duplication and endothelial injury. Ultrathin sections were prepared from epoxy-resin–embedded tissue and examined using a transmission electron microscope under standard conditions. TEM images were used for qualitative confirmation and were not included in quantitative analyses.

**Statistical analysis**

Statistical analyses were performed using JMP Pro 17 (SAS Institute), GraphPad Prism 10 (GraphPad), and R (version 4.5.2; R Foundation for Statistical Computing). Normality of continuous variables was assessed using the Shapiro–Wilk test. Between-group comparisons were performed using Student’s t-test or the Mann–Whitney U test, as appropriate. Categorical variables were compared using the χ² test or Fisher’s exact test.

Associations between clinical variables and glomerular staining intensity were evaluated using univariate and multivariable logistic regression models incorporating clinically relevant covariates, including recipient age, donor age, ABO compatibility, HLA mismatch, donor-specific antibody (DSA) status, and treatment variables. For Δ-based logistic models, a decrease in marker intensity (Δ < 0) was coded as the outcome (event), where Δ values were defined as post-treatment minus pre-treatment intensity (Δ = post − pre).

Death-censored graft survival was analyzed using Kaplan–Meier curves with log-rank testing and evaluated using multivariable Cox proportional hazards regression. Death-censored graft failure was defined as return to maintenance dialysis, with death with a functioning graft treated as censoring. For Cox models incorporating Δ values, follow-up time was defined as months from the pre-treatment (index) biopsy to death-censored graft failure or censoring.

ΔPV-1 and ΔC4d were calculated from paired pre- and post-treatment biopsies as post-treatment minus pre-treatment intensity and were entered as fixed covariates. Because Δ values were only available in patients with paired biopsies, Cox analyses incorporating Δ were restricted to this subset. Prespecified covariates for multivariable Cox models included recipient age, donor age, sex, ABO incompatibility, de novo DSA, baseline serum creatinine at the pre-treatment biopsy, steroid pulse therapy, rituximab, and intravenous immunoglobulin (IVIg). Baseline serum creatinine was selected as the renal function covariate because eGFR can be unstable and treatment-sensitive in the peri-intervention setting, whereas serum creatinine provides a more direct and comparable measure across time points. When applicable, ΔPV-1 and ΔC4d were entered simultaneously in the same model.

To account for potential non-independence of repeated biopsies within the same patient, sensitivity analyses were performed using patient-level clustering with robust standard errors. Biopsy interval (months) between paired biopsies was additionally tested as a covariate in Δ-based models. The proportional hazards assumption was assessed using Schoenfeld residuals for each covariate and for the global model. Statistical significance was defined as a two-sided P value < 0.05.

**Supplementary Figure S1. Flowchart of patient selection for the study.**

A total of 1,517 kidney transplant recipients who underwent 3,826 allograft biopsies between January 2012 and December 2022 were screened. Exclusion criteria included recurrent or de novo glomerular disease (IgAN, MN, MPGN, or lupus nephritis; 118 patients, 292 biopsies), T cell–mediated rejection (TCMR; 95 patients, 386 biopsies), BK polyomavirus infection/nephropathy (16 patients, 68 biopsies), and cases with acute antibody-mediated rejection (ABMR) without subsequent follow-up biopsy available for longitudinal evaluation (208 patients, 684 biopsies). After exclusions, 1,225 recipients (2,820 biopsies) remained eligible. The final study cohort comprised 126 patients (448 biopsies) diagnosed with chronic active ABMR according to the Banff classification.

The control cohort consisted of 94 long-term stable recipients (345 biopsies) who underwent at least three biopsies over a post-transplant period exceeding eight years. Control biopsies were rigorously reviewed and excluded if any evidence of rejection (TCMR or ABMR) or BK virus–associated nephropathy was identified.

**Supplementary Figure S2. Glomerular C4d deposition and PV-1 expression in a control case with ABO compatibility.**

Representative serial allograft biopsies from a stable control recipient with ABO-compatible transplantation. (A–C) Periodic acid–Schiff (PAS) staining; (D–F) periodic acid–methenamine silver (PAMS) staining; (G–I) C4d immunofluorescence; (J–L) PV-1 immunofluorescence. Biopsies were obtained at (A, D, G, J) 1 year 7 months, (B, E, H, K) 2 years 4 months, and (C, F, I, L) 3 years 9 months post-transplantation. C4d shows low-level glomerular endothelial/mesangial signal under standardized acquisition settings, whereas PV-1 is absent in glomeruli across all time points. Scale bars, 20 μm.

**Supplementary Figure S3. Glomerular C4d deposition and PV-1 expression in a control case with ABO incompatibility.**

Representative serial allograft biopsies from a stable control recipient with ABO-incompatible transplantation. (A–C) PAS staining; (D–F) PAMS staining; (G–I) C4d immunofluorescence; (J–L) PV-1 immunofluorescence. Biopsies were obtained at (A, D, G, J) 2 months, (B, E, H, K) 4 months, and (C, F, I, L) 1 year post-transplantation. Glomerular C4d shows background signal in this ABO-incompatible control, whereas PV-1 remains absent in glomeruli at all time points. Scale bars, 20 μm.

**Supplementary Figure S4. Representative longitudinal glomerular C4d and PV-1 immunofluorescence images in caABMR cases spanning low, intermediate, and high PV-1 intensity.**

Serial biopsies from three chronic active antibody-mediated rejection (caABMR) cases selected to illustrate the spectrum of glomerular PV-1 intensity (Case 1, low; Case 2, intermediate; Case 3, high) and longitudinal changes following anti-rejection therapies. For each case, C4d immunofluorescence is shown in the upper row and PV-1 immunofluorescence in the lower row. Dashed lines delineate the glomerular tuft region of interest (ROI) used for quantitative intensity measurements.

Case 1: (A, D) pre-treatment (no anti-rejection therapy administered before this biopsy); (B, E) after methylprednisolone pulse therapy plus rituximab; (C, F) after IVIg.

Case 2: (G, J) immediately after combined therapy (prednisolone, rituximab, and IVIg); (H, K) follow-up biopsy without additional new agents; (I, L) after the second rituximab administration.

Case 3: (M, P) pre-treatment (no anti-rejection therapy administered before this biopsy); (N, Q) after combined therapy (prednisolone, rituximab, and IVIg); (O, R) after the second IVIg course. Scale bars, 20 μm.

**Supplementary Figure S5. Glomerular C4d deposition and PV-1 expression in relation to clinical and histological factors in chronic active antibody-mediated rejection.**

Cases of chronic active antibody-mediated rejection (cABMR) were stratified by the presence or absence of microvascular inflammation (MVI), defined as a combined Banff 2022 glomerulitis (g) and peritubular capillaritis (ptc) score ≥ 2, and compared with control biopsies. Glomerular C4d deposition and PV-1 intensity were analyzed across the following contexts: (A, B) biopsies obtained within 1 year versus more than 1 year after transplantation; (C, D) ABO-compatible versus ABO-incompatible kidney transplants; (E, F) presence versus absence of donor-specific antibodies (DSA); (G, H) presence versus absence of diabetes mellitus (DM). Data are presented as mean ± standard deviation, with P values from intergroup comparisons indicated in each panel.

**Supplementary Figure S6. Survival curves stratified by treatment modalities in chronic active antibody-mediated rejection.**

Kaplan–Meier survival curves comparing patients with and without treatment using: (A) methylprednisolone (mPSL) pulse therapy, (B) double filtration plasmapheresis (DFPP), (C) deoxyspergualin (DSG), and (D) intravenous immunoglobulin (IVIg).

**Supplementary Figure S7. Multivariable logistic regression analyses of clinical factors associated with within-patient changes (Δ) in glomerular C4d and PV-1 intensity.**

Forest plots show odds ratios (ORs) and 95% confidence intervals (CIs) from multivariable logistic regression models evaluating clinical covariates associated with a decrease in glomerular marker intensity after treatment. Δ was defined as post-treatment minus pre-treatment intensity (Δ = post − pre). For these analyses, a decrease in intensity (Δ < 0) was coded as the outcome (event). (A) Overall cohort for ΔC4d. (B) Overall cohort for ΔPV-1. (C, D) ABO-compatible subgroup: ΔC4d (C) and ΔPV-1 (D). (E, F) ABO-incompatible subgroup: ΔC4d (E) and ΔPV-1 (F). Red markers indicate covariates with P < 0.05.

**Supplementary Figure S8. Allograft survival according to dynamic changes in glomerular C4d and PV-1 intensity stratified by ABO compatibility.**

Kaplan–Meier curves show death-censored graft survival stratified by within-patient changes (Δ) in glomerular C4d and plasmalemma vesicle-associated protein-1 (PV-1) intensity after treatment. (A) ABO-compatible transplants stratified by tertiles of ΔC4d. (B) ABO-compatible transplants stratified by tertiles of ΔPV-1. (C) ABO-incompatible transplants stratified by tertiles of ΔC4d. (D) ABO-incompatible transplants stratified by tertiles of ΔPV-1. ΔC4d and ΔPV-1 were defined as post-treatment minus pre-treatment intensity (Δ = post − pre). Patients were categorized into tertiles (low, middle, high) according to the magnitude of Δ values.

**Supplementary Figure S9. Death-censored graft survival according to dynamic changes in glomerular C4d and PV-1 intensity stratified by rituximab exposure.**

Kaplan–Meier curves show death-censored graft survival stratified by within-patient changes (Δ) in glomerular C4d and plasmalemma vesicle-associated protein-1 (PV-1) intensity after treatment, with subgrouping by rituximab exposure. (A) Survival stratified by ΔC4d groups in patients treated with or without rituximab. (B) Survival stratified by ΔPV-1 groups in patients treated with or without rituximab. ΔC4d and ΔPV-1 were defined as post-treatment minus pre-treatment intensity (Δ = post − pre). P values were calculated using the log-rank test across the four groups.

**Supplementary Figure S10. Sensitivity multivariable Cox proportional hazards model (Model 2) incorporating ΔPV-1 and ΔC4d for death-censored graft failure.**

Forest plot of hazard ratios (HRs) and 95% confidence intervals (CIs) from a prespecified multivariable Cox proportional hazards model including both ΔPV-1 and ΔC4d, defined as within-patient changes in glomerular staining intensity between paired biopsies (post-treatment minus pre-treatment; post − pre). The model additionally adjusted for recipient age, donor age, sex, ABO incompatibility, de novo donor-specific antibody (DSA), diabetes, baseline serum creatinine at the pre-treatment (index) biopsy, methylprednisolone (mPSL) pulse therapy, rituximab, intravenous immunoglobulin (IVIg), and the number of HLA mismatches. Time-to-event was calculated from the pre-treatment (index) biopsy to death-censored graft failure (return to maintenance dialysis); patients without the event were censored at the last follow-up. HRs are displayed on a logarithmic scale. Variables with P < 0.05 are highlighted in red.

**Supplementary Table S1. Multivariable Cox proportional hazards models and proportional hazards diagnostics (Schoenfeld residual tests).**

The table summarizes hazard ratios (HRs), 95% confidence intervals (CIs), and P values from multivariable Cox proportional hazards models evaluating death-censored graft survival in chronic active antibody-mediated rejection (caABMR). Follow-up time was defined as months from the pre-treatment (index) biopsy at caABMR diagnosis to death-censored graft failure (return to maintenance dialysis); patients without the event were censored at the last follow-up. Death with a functioning graft was treated as censoring.

ΔPV-1 and ΔC4d were defined as within-patient changes in glomerular staining intensity between paired pre- and post-treatment biopsies (post − pre) and were entered as fixed covariates. Analyses including Δ variables were restricted to patients with paired biopsies, and Δ covariates were interpreted as longitudinal response measures anchored to the index biopsy.

Model 1 included ΔPV-1 and prespecified clinical covariates: recipient age, donor age, sex, ABO incompatibility, de novo donor-specific antibody (DSA), diabetes, baseline serum creatinine at the pre-treatment biopsy, methylprednisolone (mPSL) pulse therapy, rituximab, intravenous immunoglobulin (IVIg), and the number of HLA mismatches. Model 2 included both ΔPV-1 and ΔC4d simultaneously with the same covariates.

Proportional hazards assumptions were evaluated using Schoenfeld residual tests for each covariate and for the global model. Test statistics (χ²), degrees of freedom (df), and P values are reported. Global P values reflect overall model-level proportionality. Given the sensitivity of Schoenfeld tests in multivariable settings, these diagnostics were interpreted in conjunction with visual inspection of scaled Schoenfeld residual plots.

Although the global Schoenfeld test for Model 2 reached nominal statistical significance, visual inspection of scaled Schoenfeld residual plots did not demonstrate a consistent time-dependent pattern for key covariates.
