# Supplementary material for: Glomerular plasmalemma vesicle‐associated protein‐1 as an endothelial remodelling marker complementing C4d in chronic active antibody‐mediated rejection
Source: Histopathology. 2026 Apr 21;89(2):372–82. doi: 10.1111/his.70162 (PMC13341019; doi:10.1111/his.70162)
Supplement: Supplementary file 2 — Table S1. Multivariable Cox proportional hazards models and proportional hazards diagnostics (Schoenfeld residual tests). [file HIS-89-372-s001.zip › his70162-sup-0015-TableS1@Supplementary Table S1.docx]

**Supplementary Table S1. Multivariable Cox proportional hazards models and proportional hazards diagnostics (Schoenfeld residual tests).**

The table summarizes hazard ratios (HRs), 95% confidence intervals (CIs), and P values from multivariable Cox proportional hazards models evaluating death-censored graft survival in chronic active antibody-mediated rejection (caABMR). Follow-up time was defined as months from the pre-treatment (index) biopsy at caABMR diagnosis to death-censored graft failure (return to maintenance dialysis); patients without the event were censored at the last follow-up. Death with a functioning graft was treated as censoring.

ΔPV-1 and ΔC4d were defined as within-patient changes in glomerular staining intensity between paired pre- and post-treatment biopsies (post − pre) and were entered as fixed covariates. Analyses including Δ variables were restricted to patients with paired biopsies, and Δ covariates were interpreted as longitudinal response measures anchored to the index biopsy.

Model 1 included ΔPV-1 and prespecified clinical covariates: recipient age, donor age, sex, ABO incompatibility, de novo donor-specific antibody (DSA), diabetes, baseline serum creatinine at the pre-treatment biopsy, methylprednisolone (mPSL) pulse therapy, rituximab, intravenous immunoglobulin (IVIg), and the number of HLA mismatches. Model 2 included both ΔPV-1 and ΔC4d simultaneously with the same covariates.

Proportional hazards assumptions were evaluated using Schoenfeld residual tests for each covariate and for the global model. Test statistics (χ²), degrees of freedom (df), and P values are reported. Global P values reflect overall model-level proportionality. Given the sensitivity of Schoenfeld tests in multivariable settings, these diagnostics were interpreted in conjunction with visual inspection of scaled Schoenfeld residual plots.

Although the global Schoenfeld test for Model 2 reached nominal statistical significance, visual inspection of scaled Schoenfeld residual plots did not demonstrate a consistent time-dependent pattern for key covariates.
