# Supplementary material for: Glomerular plasmalemma vesicle‐associated protein‐1 as an endothelial remodelling marker complementing C4d in chronic active antibody‐mediated rejection
Source: Histopathology. 2026 Apr 21;89(2):372–82. doi: 10.1111/his.70162 (PMC13341019; doi:10.1111/his.70162)
Supplement: Supplementary file 2 — Table S1. Multivariable Cox proportional hazards models and proportional hazards diagnostics (Schoenfeld residual tests). [file HIS-89-372-s001.zip › HIS_70162__author.docx]

| Model | Term | Chisq | Df | P-value |
| --- | --- | --- | --- | --- |
| Model　1 | ΔPV-1 (post - pre) | 1.50 | 1 | 0.221 |
| Model　1 | Recipient age (years) | 3.04 | 1 | 0.081 |
| Model　1 | Donor age (years) | 0.03 | 1 | 0.854 |
| Model　1 | Sex (male) | 0.10 | 1 | 0.751 |
| Model　1 | ABO incompatible | 0.37 | 1 | 0.545 |
| Model　1 | De novo DSA | 0.18 | 1 | 0.669 |
| Model　1 | Baseline serum creatinine (mg/dL) | 0.00 | 1 | 0.977 |
| Model　1 | mPSL pulse therapy | 0.51 | 1 | 0.474 |
| Model　1 | Rituximab | 0.52 | 1 | 0.471 |
| Model　1 | IVIg | 1.32 | 1 | 0.250 |
| Model　1 | Number of HLA mismatches | 0.12 | 1 | 0.724 |
| Model　1 | Diabetes | 0.56 | 1 | 0.454 |
| Model　1 | GLOBAL | 18.44 | 12 | 0.103 |
| Model　2 | ΔPV-1 (post - pre) | 1.11 | 1 | 0.292 |
| Model　2 | ΔC4d (post - pre) | 4.42 | 1 | 0.035 |
| Model　2 | Recipient age (years) | 2.91 | 1 | 0.088 |
| Model　2 | Donor age (years) | 0.06 | 1 | 0.811 |
| Model　2 | Sex (male) | 0.04 | 1 | 0.846 |
| Model　2 | ABO incompatible | 0.45 | 1 | 0.502 |
| Model　2 | De novo DSA | 0.52 | 1 | 0.471 |
| Model　2 | Baseline serum creatinine (mg/dL) | 0.00 | 1 | 0.979 |
| Model　2 | mPSL pulse therapy | 0.47 | 1 | 0.491 |
| Model　2 | Rituximab | 1.45 | 1 | 0.229 |
| Model　2 | IVIg | 0.90 | 1 | 0.343 |
| Model　2 | Number of HLA mismatches | 0.12 | 1 | 0.733 |
| Model　2 | Diabetes | 0.71 | 1 | 0.399 |
| Model　2 | GLOBAL | 23.12 | 13 | 0.400 |
